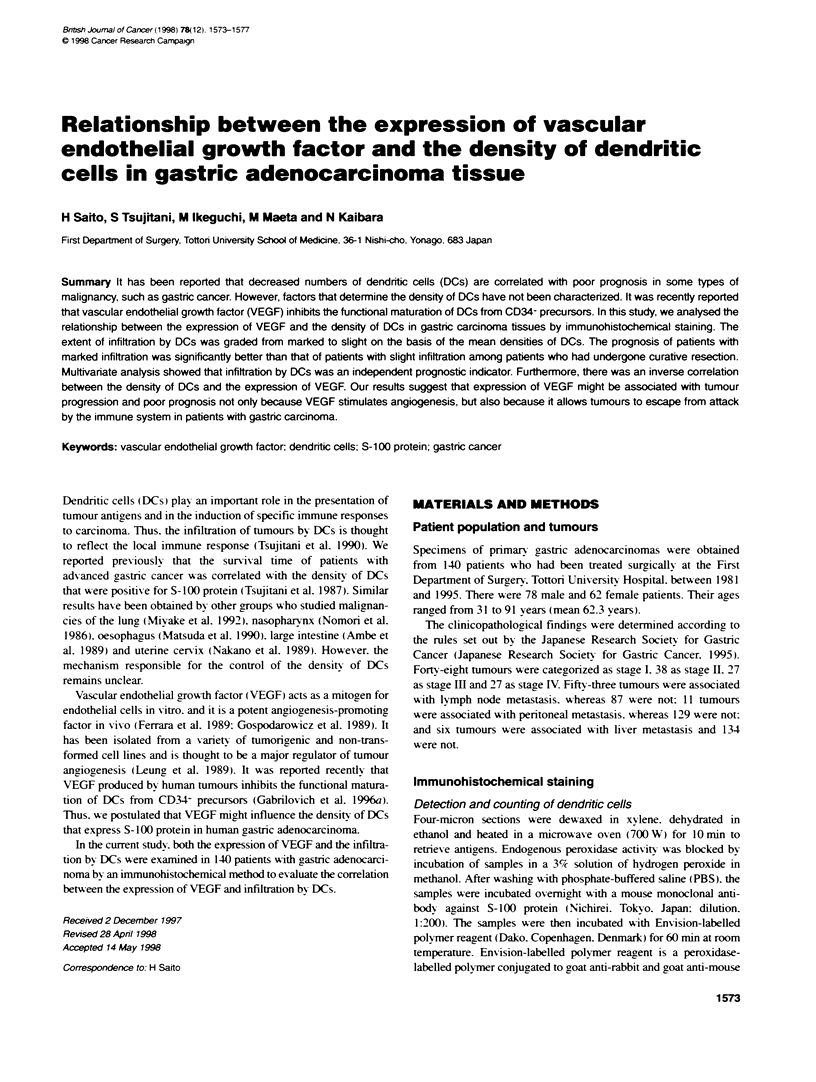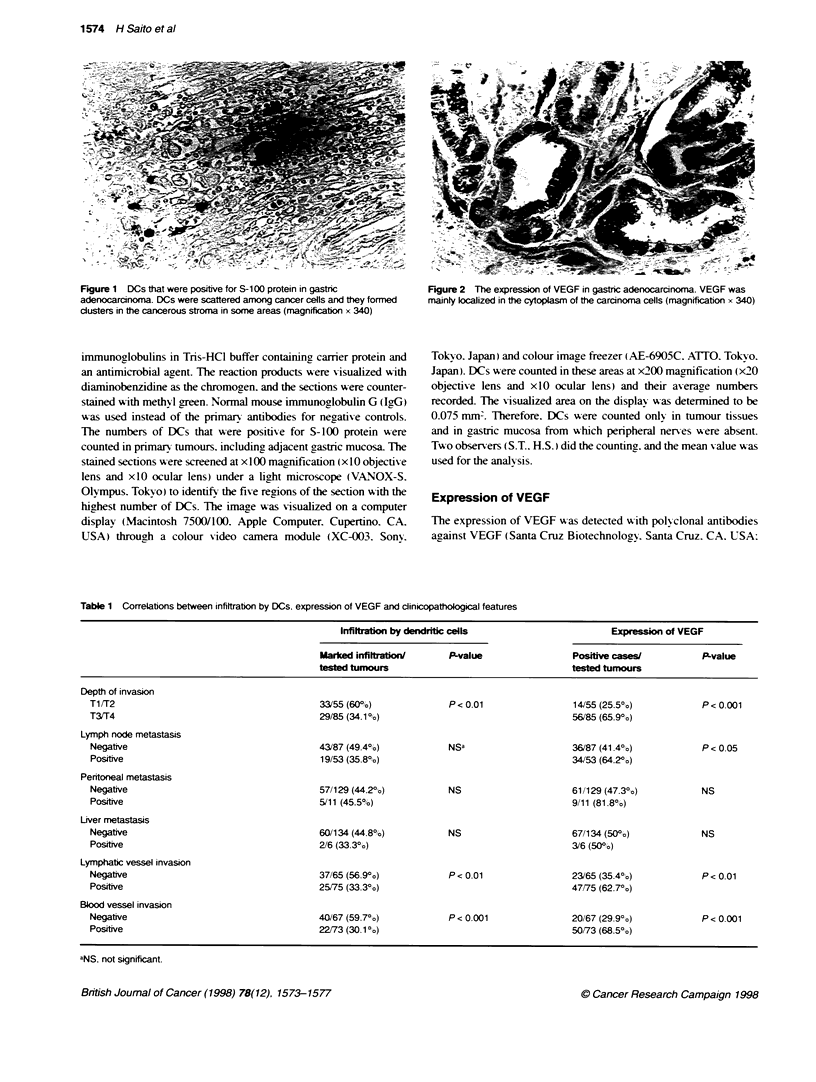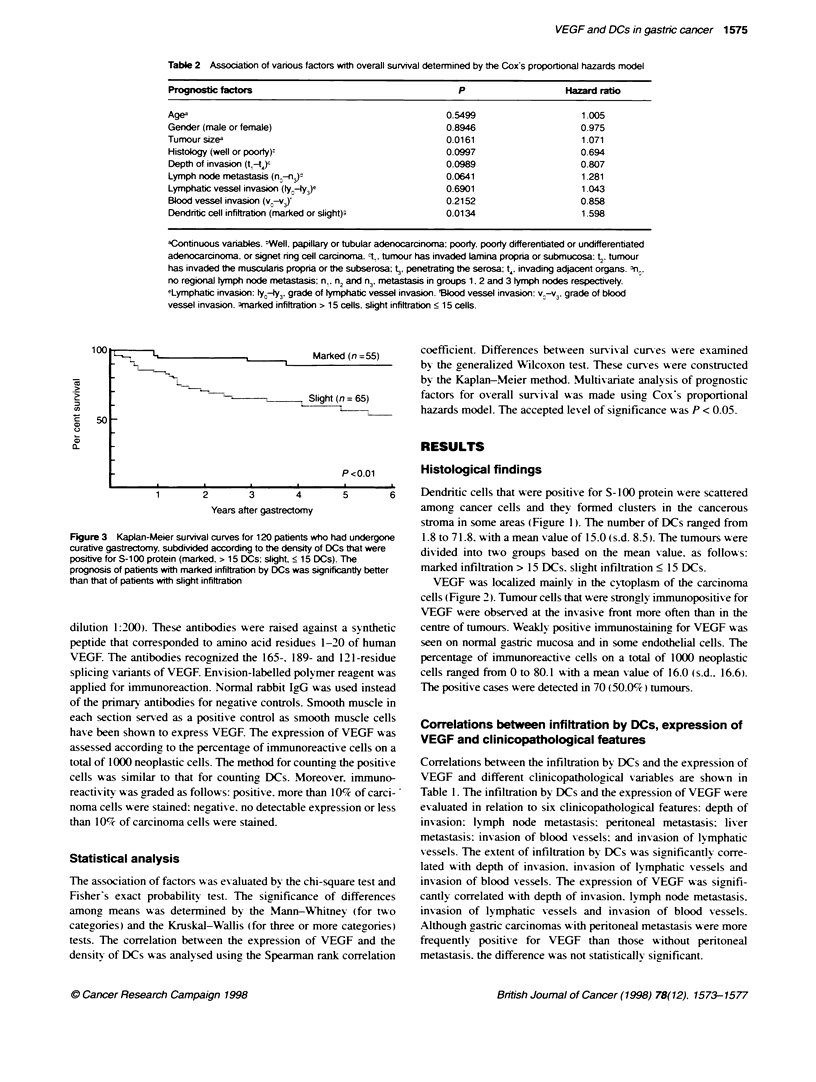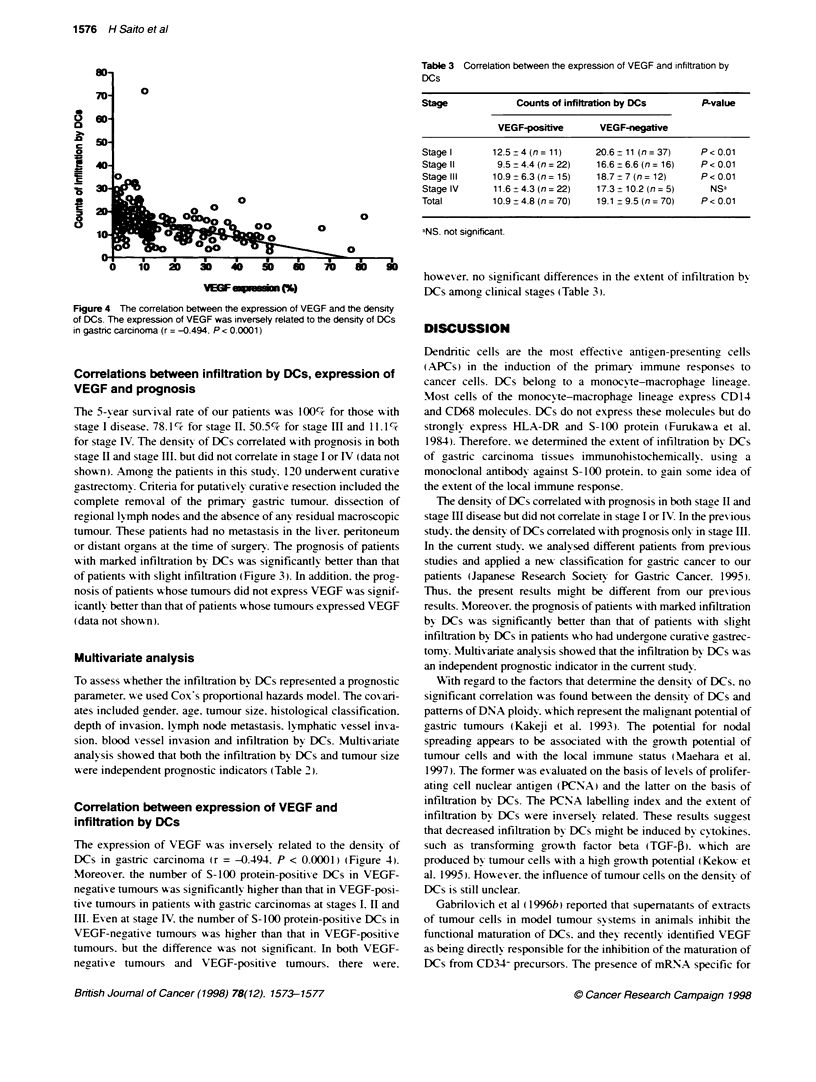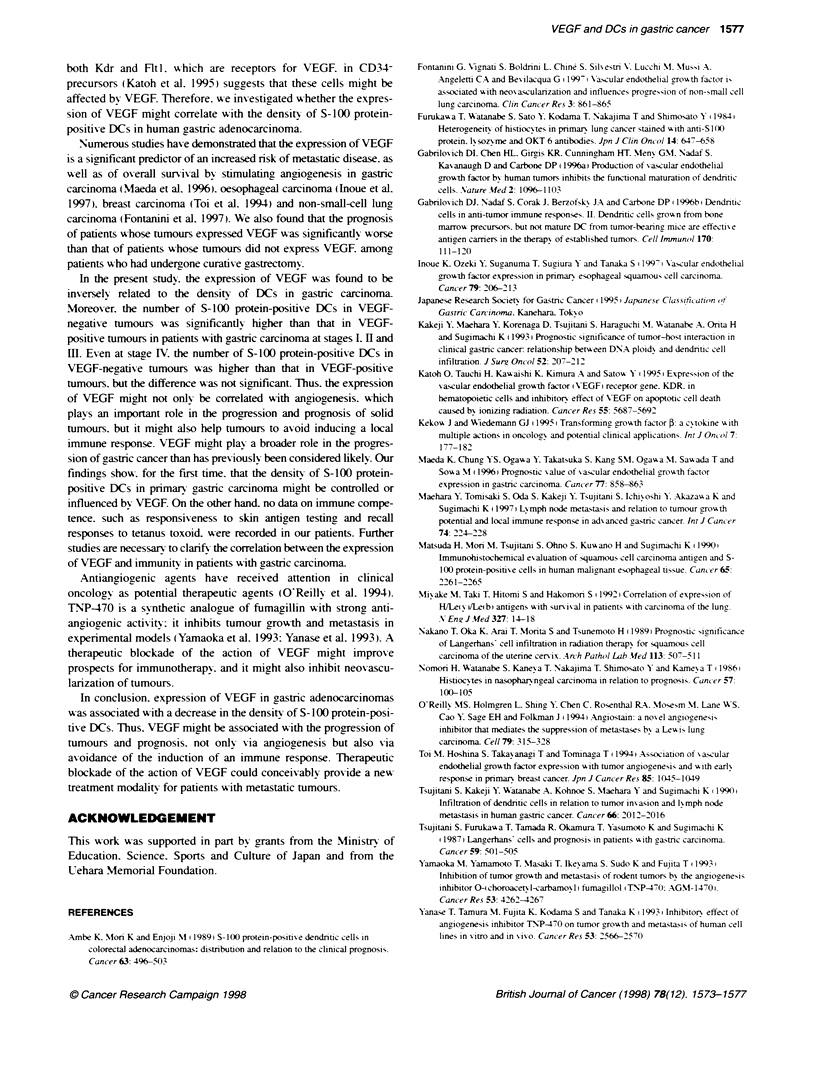# Relationship between the expression of vascular endothelial growth factor and the density of dendritic cells in gastric adenocarcinoma tissue.

**DOI:** 10.1038/bjc.1998.725

**Published:** 1998-12

**Authors:** H. Saito, S. Tsujitani, M. Ikeguchi, M. Maeta, N. Kaibara

**Affiliations:** First Department of Surgery, Tottori University School of Medicine, Yonago, Japan.

## Abstract

**Images:**


British Journal of Cancer (1998) 78(12), 1573–1577
© 1998 Cancer Research Campaign

### Relationship between the expression of vascular endothelial growth factor and the density of dendritic cells in gastric adenocarcinoma tissue

**H Saito, S Tsujitani, M Ikeguchi, M Maeta and N Kaibara**

First Department of Surgery, Tottori University School of Medicine, 36-1 Nishi-cho, Yonago, 683 Japan

**Summary** It has been reported that decreased numbers of dendritic cells (DCs) are correlated with poor prognosis in some types of malignancy, such as gastric cancer. However, factors that determine the density of DCs have not been characterized. It was recently reported that vascular endothelial growth factor (VEGF) inhibits the functional maturation of DCs from $CD34^+$ precursors. In this study, we analysed the relationship between the expression of VEGF and the density of DCs in gastric carcinoma tissues by immunohistochemical staining. The extent of infiltration by DCs was graded from marked to slight on the basis of the mean densities of DCs. The prognosis of patients with marked infiltration was significantly better than that of patients with slight infiltration among patients who had undergone curative resection. Multivariate analysis showed that infiltration by DCs was an independent prognostic indicator. Furthermore, there was an inverse correlation between the density of DCs and the expression of VEGF. Our results suggest that expression of VEGF might be associated with tumour progression and poor prognosis not only because VEGF stimulates angiogenesis, but also because it allows tumours to escape from attack by the immune system in patients with gastric carcinoma.

**Keywords:** vascular endothelial growth factor; dendritic cells; S-100 protein; gastric cancer

Dendritic cells (DCs) play an important role in the presentation of tumour antigens and in the induction of specific immune responses to carcinoma. Thus, the infiltration of tumours by DCs is thought to reflect the local immune response (Tsujitani et al, 1990). We reported previously that the survival time of patients with advanced gastric cancer was correlated with the density of DCs that were positive for S-100 protein (Tsujitani et al, 1987). Similar results have been obtained by other groups who studied malignancies of the lung (Miyake et al, 1992), nasopharynx (Nomori et al, 1986), oesophagus (Matsuda et al, 1990), large intestine (Ambe et al, 1989) and uterine cervix (Nakano et al, 1989). However, the mechanism responsible for the control of the density of DCs remains unclear.

Vascular endothelial growth factor (VEGF) acts as a mitogen for endothelial cells in vitro, and it is a potent angiogenesis-promoting factor in vivo (Ferrara et al, 1989; Gospodarowicz et al, 1989). It has been isolated from a variety of tumorigenic and non-transformed cell lines and is thought to be a major regulator of tumour angiogenesis (Leung et al, 1989). It was reported recently that VEGF produced by human tumours inhibits the functional maturation of DCs from $CD34^+$ precursors (Gabrilovich et al, 1996*a*). Thus, we postulated that VEGF might influence the density of DCs that express S-100 protein in human gastric adenocarcinoma.

In the current study, both the expression of VEGF and the infiltration by DCs were examined in 140 patients with gastric adenocarcinoma by an immunohistochemical method to evaluate the correlation between the expression of VEGF and infiltration by DCs.

*Received 2 December 1997*

*Revised 28 April 1998*

*Accepted 14 May 1998*

*Correspondence to:* H Saito

### MATERIALS AND METHODS

### Patient population and tumours

Specimens of primary gastric adenocarcinomas were obtained from 140 patients who had been treated surgically at the First Department of Surgery, Tottori University Hospital, between 1981 and 1995. There were 78 male and 62 female patients. Their ages ranged from 31 to 91 years (mean 62.3 years).

The clinicopathological findings were determined according to the rules set out by the Japanese Research Society for Gastric Cancer (Japanese Research Society for Gastric Cancer, 1995). Forty-eight tumours were categorized as stage I, 38 as stage II, 27 as stage III and 27 as stage IV. Fifty-three tumours were associated with lymph node metastasis, whereas 87 were not; 11 tumours were associated with peritoneal metastasis, whereas 129 were not; and six tumours were associated with liver metastasis and 134 were not.

### Immunohistochemical staining

*Detection and counting of dendritic cells*

Four-micron sections were dewaxed in xylene, dehydrated in ethanol and heated in a microwave oven (700 W) for 10 min to retrieve antigens. Endogenous peroxidase activity was blocked by incubation of samples in a 3% solution of hydrogen peroxide in methanol. After washing with phosphate-buffered saline (PBS), the samples were incubated overnight with a mouse monoclonal antibody against S-100 protein (Nichirei, Tokyo, Japan; dilution, 1:200). The samples were then incubated with Envision-labelled polymer reagent (Dako, Copenhagen, Denmark) for 60 min at room temperature. Envision-labelled polymer reagent is a peroxidase-labelled polymer conjugated to goat anti-rabbit and goat anti-mouse

**Figure 1** DCs that were positive for S-100 protein in gastric adenocarcinoma. DCs were scattered among cancer cells and they formed clusters in the cancerous stroma in some areas (magnification × 340)

**Figure 2** The expression of VEGF in gastric adenocarcinoma. VEGF was mainly localized in the cytoplasm of the carcinoma cells (magnification × 340)

immunoglobulins in Tris-HCl buffer containing carrier protein and an antimicrobial agent. The reaction products were visualized with diaminobenzidine as the chromogen, and the sections were counter-stained with methyl green. Normal mouse immunoglobulin G (IgG) was used instead of the primary antibodies for negative controls. The numbers of DCs that were positive for S-100 protein were counted in primary tumours, including adjacent gastric mucosa. The stained sections were screened at ×100 magnification (×10 objective lens and ×10 ocular lens) under a light microscope (VANOX-S, Olympus, Tokyo) to identify the five regions of the section with the highest number of DCs. The image was visualized on a computer display (Macintosh 7500/100, Apple Computer, Cupertino, CA, USA) through a colour video camera module (XC-003, Sony, Tokyo, Japan) and colour image freezer (AE-6905C, ATTO, Tokyo, Japan). DCs were counted in these areas at ×200 magnification (×20 objective lens and ×10 ocular lens) and their average numbers recorded. The visualized area on the display was determined to be 0.075 $mm^2$. Therefore, DCs were counted only in tumour tissues and in gastric mucosa from which peripheral nerves were absent. Two observers (S.T., H.S.) did the counting, and the mean value was used for the analysis.

### Expression of VEGF

The expression of VEGF was detected with polyclonal antibodies against VEGF (Santa Cruz Biotechnology, Santa Cruz, CA, USA;

**Table 1** Correlations between infiltration by DCs, expression of VEGF and clinicopathological features

| | Infiltration by dendritic cells | | Expression of VEGF | |
|---|---|---|---|---|
| | Marked infiltration/ tested tumours | P-value | Positive cases/ tested tumours | P-value |
| Depth of invasion | | | | |
| T1/T2 | 33/55 (60%) | P < 0.01 | 14/55 (25.5%) | P < 0.001 |
| T3/T4 | 29/85 (34.1%) | | 56/85 (65.9%) | |
| Lymph node metastasis | | | | |
| Negative | 43/87 (49.4%) | NS[a] | 36/87 (41.4%) | P < 0.05 |
| Positive | 19/53 (35.8%) | | 34/53 (64.2%) | |
| Peritoneal metastasis | | | | |
| Negative | 57/129 (44.2%) | NS | 61/129 (47.3%) | NS |
| Positive | 5/11 (45.5%) | | 9/11 (81.8%) | |
| Liver metastasis | | | | |
| Negative | 60/134 (44.8%) | NS | 67/134 (50%) | NS |
| Positive | 2/6 (33.3%) | | 3/6 (50%) | |
| Lymphatic vessel invasion | | | | |
| Negative | 37/65 (56.9%) | P < 0.01 | 23/65 (35.4%) | P < 0.01 |
| Positive | 25/75 (33.3%) | | 47/75 (62.7%) | |
| Blood vessel invasion | | | | |
| Negative | 40/67 (59.7%) | P < 0.001 | 20/67 (29.9%) | P < 0.001 |
| Positive | 22/73 (30.1%) | | 50/73 (68.5%) | |

[a]NS, not significant.

© Cancer Research Campaign 1998

**Table 2** Association of various factors with overall survival determined by the Cox's proportional hazards model

| Prognostic factors | *P* | Hazard ratio |
|---|---|---|
| Age[a] | 0.5499 | 1.005 |
| Gender (male or female) | 0.8946 | 0.975 |
| Tumour size[a] | 0.0161 | 1.071 |
| Histology (well or poorly)[b] | 0.0997 | 0.694 |
| Depth of invasion ($t_1$–$t_4$)[c] | 0.0989 | 0.807 |
| Lymph node metastasis ($n_0$–$n_3$)[d] | 0.0641 | 1.281 |
| Lymphatic vessel invasion ($ly_0$–$ly_3$)[e] | 0.6901 | 1.043 |
| Blood vessel invasion ($v_0$–$v_3$)[f] | 0.2152 | 0.858 |
| Dendritic cell infiltration (marked or slight)[g] | 0.0134 | 1.598 |

[a]Continuous variables. [b]Well, papillary or tubular adenocarcinoma; poorly, poorly differentiated or undifferentiated adenocarcinoma, or signet ring cell carcinoma. [c]$t_1$, tumour has invaded lamina propria or submucosa; $t_2$, tumour has invaded the muscularis propria or the subserosa; $t_3$, penetrating the serosa; $t_4$, invading adjacent organs. [d]$n_0$, no regional lymph node metastasis; $n_1$, $n_2$ and $n_3$, metastasis in groups 1, 2 and 3 lymph nodes respectively. [e]Lymphatic invasion: $ly_0$–$ly_3$, grade of lymphatic vessel invasion. [f]Blood vessel invasion: $v_0$–$v_3$, grade of blood vessel invasion. [g]marked infiltration > 15 cells, slight infiltration ≤ 15 cells.

**Figure 3** Kaplan-Meier survival curves for 120 patients who had undergone curative gastrectomy, subdivided according to the density of DCs that were positive for S-100 protein (marked, > 15 DCs; slight, ≤ 15 DCs). The prognosis of patients with marked infiltration by DCs was significantly better than that of patients with slight infiltration dilution 1:200). These antibodies were raised against a synthetic peptide that corresponded to amino acid residues 1–20 of human VEGF. The antibodies recognized the 165-, 189- and 121-residue splicing variants of VEGF. Envision-labelled polymer reagent was applied for immunoreaction. Normal rabbit IgG was used instead of the primary antibodies for negative controls. Smooth muscle in each section served as a positive control as smooth muscle cells have been shown to express VEGF. The expression of VEGF was assessed according to the percentage of immunoreactive cells on a total of 1000 neoplastic cells. The method for counting the positive cells was similar to that for counting DCs. Moreover, immunoreactivity was graded as follows: positive, more than 10% of carcinoma cells were stained; negative, no detectable expression or less than 10% of carcinoma cells were stained.

### Statistical analysis

The association of factors was evaluated by the chi-square test and Fisher's exact probability test. The significance of differences among means was determined by the Mann–Whitney (for two categories) and the Kruskal–Wallis (for three or more categories) tests. The correlation between the expression of VEGF and the density of DCs was analysed using the Spearman rank correlation coefficient. Differences between survival curves were examined by the generalized Wilcoxon test. These curves were constructed by the Kaplan–Meier method. Multivariate analysis of prognostic factors for overall survival was made using Cox's proportional hazards model. The accepted level of significance was $P < 0.05$.

### RESULTS

### Histological findings

Dendritic cells that were positive for S-100 protein were scattered among cancer cells and they formed clusters in the cancerous stroma in some areas (Figure 1). The number of DCs ranged from 1.8 to 71.8, with a mean value of 15.0 (s.d. 8.5). The tumours were divided into two groups based on the mean value, as follows: marked infiltration > 15 DCs, slight infiltration ≤ 15 DCs.

VEGF was localized mainly in the cytoplasm of the carcinoma cells (Figure 2). Tumour cells that were strongly immunopositive for VEGF were observed at the invasive front more often than in the centre of tumours. Weakly positive immunostaining for VEGF was seen on normal gastric mucosa and in some endothelial cells. The percentage of immunoreactive cells on a total of 1000 neoplastic cells ranged from 0 to 80.1 with a mean value of 16.0 (s.d., 16.6). The positive cases were detected in 70 (50.0%) tumours.

### Correlations between infiltration by DCs, expression of VEGF and clinicopathological features

Correlations between the infiltration by DCs and the expression of VEGF and different clinicopathological variables are shown in Table 1. The infiltration by DCs and the expression of VEGF were evaluated in relation to six clinicopathological features: depth of invasion; lymph node metastasis; peritoneal metastasis; liver metastasis; invasion of blood vessels; and invasion of lymphatic vessels. The extent of infiltration by DCs was significantly correlated with depth of invasion, invasion of lymphatic vessels and invasion of blood vessels. The expression of VEGF was significantly correlated with depth of invasion, lymph node metastasis, invasion of lymphatic vessels and invasion of blood vessels. Although gastric carcinomas with peritoneal metastasis were more frequently positive for VEGF than those without peritoneal metastasis, the difference was not statistically significant.

© Cancer Research Campaign 1998

**Figure 4** The correlation between the expression of VEGF and the density of DCs. The expression of VEGF was inversely related to the density of DCs in gastric carcinoma (r = –0.494, $P < 0.0001$)

### Correlations between infiltration by DCs, expression of VEGF and prognosis

The 5-year survival rate of our patients was 100% for those with stage I disease, 78.1% for stage II, 50.5% for stage III and 11.1% for stage IV. The density of DCs correlated with prognosis in both stage II and stage III, but did not correlate in stage I or IV (data not shown). Among the patients in this study, 120 underwent curative gastrectomy. Criteria for putatively curative resection included the complete removal of the primary gastric tumour, dissection of regional lymph nodes and the absence of any residual macroscopic tumour. These patients had no metastasis in the liver, peritoneum or distant organs at the time of surgery. The prognosis of patients with marked infiltration by DCs was significantly better than that of patients with slight infiltration (Figure 3). In addition, the prognosis of patients whose tumours did not express VEGF was significantly better than that of patients whose tumours expressed VEGF (data not shown).

### Multivariate analysis

To assess whether the infiltration by DCs represented a prognostic parameter, we used Cox's proportional hazards model. The covariates included gender, age, tumour size, histological classification, depth of invasion, lymph node metastasis, lymphatic vessel invasion, blood vessel invasion and infiltration by DCs. Multivariate analysis showed that both the infiltration by DCs and tumour size were independent prognostic indicators (Table 2).

### Correlation between expression of VEGF and infiltration by DCs

The expression of VEGF was inversely related to the density of DCs in gastric carcinoma (r = –0.494, $P < 0.0001$) (Figure 4). Moreover, the number of S-100 protein-positive DCs in VEGF-negative tumours was significantly higher than that in VEGF-positive tumours in patients with gastric carcinomas at stages I, II and III. Even at stage IV, the number of S-100 protein-positive DCs in VEGF-negative tumours was higher than that in VEGF-positive tumours, but the difference was not significant. In both VEGF-negative tumours and VEGF-positive tumours, there were, however, no significant differences in the extent of infiltration by DCs among clinical stages (Table 3).

**Table 3** Correlation between the expression of VEGF and infiltration by DCs

| Stage | Counts of infiltration by DCs | | P-value |
|---|---|---|---|
| | VEGF-positive | VEGF-negative | |
| Stage I | 12.5 ± 4 ($n$ = 11) | 20.6 ± 11 ($n$ = 37) | $P < 0.01$ |
| Stage II | 9.5 ± 4.4 ($n$ = 22) | 16.6 ± 6.6 ($n$ = 16) | $P < 0.01$ |
| Stage III | 10.9 ± 6.3 ($n$ = 15) | 18.7 ± 7 ($n$ = 12) | $P < 0.01$ |
| Stage IV | 11.6 ± 4.3 ($n$ = 22) | 17.3 ± 10.2 ($n$ = 5) | NS[a] |
| Total | 10.9 ± 4.8 ($n$ = 70) | 19.1 ± 9.5 ($n$ = 70) | $P < 0.01$ |

[a]NS, not significant.

### DISCUSSION

Dendritic cells are the most effective antigen-presenting cells (APCs) in the induction of the primary immune responses to cancer cells. DCs belong to a monocyte–macrophage lineage. Most cells of the monocyte–macrophage lineage express CD14 and CD68 molecules. DCs do not express these molecules but do strongly express HLA-DR and S-100 protein (Furukawa et al, 1984). Therefore, we determined the extent of infiltration by DCs of gastric carcinoma tissues immunohistochemically, using a monoclonal antibody against S-100 protein, to gain some idea of the extent of the local immune response.

The density of DCs correlated with prognosis in both stage II and stage III disease but did not correlate in stage I or IV. In the previous study, the density of DCs correlated with prognosis only in stage III. In the current study, we analysed different patients from previous studies and applied a new classification for gastric cancer to our patients (Japanese Research Society for Gastric Cancer, 1995). Thus, the present results might be different from our previous results. Moreover, the prognosis of patients with marked infiltration by DCs was significantly better than that of patients with slight infiltration by DCs in patients who had undergone curative gastrectomy. Multivariate analysis showed that the infiltration by DCs was an independent prognostic indicator in the current study.

With regard to the factors that determine the density of DCs, no significant correlation was found between the density of DCs and patterns of DNA ploidy, which represent the malignant potential of gastric tumours (Kakeji et al, 1993). The potential for nodal spreading appears to be associated with the growth potential of tumour cells and with the local immune status (Maehara et al, 1997). The former was evaluated on the basis of levels of proliferating cell nuclear antigen (PCNA) and the latter on the basis of infiltration by DCs. The PCNA labelling index and the extent of infiltration by DCs were inversely related. These results suggest that decreased infiltration by DCs might be induced by cytokines, such as transforming growth factor beta (TGF-β), which are produced by tumour cells with a high growth potential (Kekow et al, 1995). However, the influence of tumour cells on the density of DCs is still unclear.

Gabrilovich et al (1996*b*) reported that supernatants of extracts of tumour cells in model tumour systems in animals inhibit the functional maturation of DCs, and they recently identified VEGF as being directly responsible for the inhibition of the maturation of DCs from $CD34^-$ precursors. The presence of mRNA specific for

© Cancer Research Campaign 1998

both Kdr and Flt1, which are receptors for VEGF, in CD34+ precursors (Katoh et al, 1995) suggests that these cells might be affected by VEGF. Therefore, we investigated whether the expression of VEGF might correlate with the density of S-100 protein-positive DCs in human gastric adenocarcinoma.

Numerous studies have demonstrated that the expression of VEGF is a significant predictor of an increased risk of metastatic disease, as well as of overall survival by stimulating angiogenesis in gastric carcinoma (Maeda et al, 1996), oesophageal carcinoma (Inoue et al, 1997), breast carcinoma (Toi et al, 1994) and non-small-cell lung carcinoma (Fontanini et al, 1997). We also found that the prognosis of patients whose tumours expressed VEGF was significantly worse than that of patients whose tumours did not express VEGF, among patients who had undergone curative gastrectomy.

In the present study, the expression of VEGF was found to be inversely related to the density of DCs in gastric carcinoma. Moreover, the number of S-100 protein-positive DCs in VEGF-negative tumours was significantly higher than that in VEGF-positive tumours in patients with gastric carcinoma at stages I, II and III. Even at stage IV, the number of S-100 protein-positive DCs in VEGF-negative tumours was higher than that in VEGF-positive tumours, but the difference was not significant. Thus, the expression of VEGF might not only be correlated with angiogenesis, which plays an important role in the progression and prognosis of solid tumours, but it might also help tumours to avoid inducing a local immune response. VEGF might play a broader role in the progression of gastric cancer than has previously been considered likely. Our findings show, for the first time, that the density of S-100 protein-positive DCs in primary gastric carcinoma might be controlled or influenced by VEGF. On the other hand, no data on immune competence, such as responsiveness to skin antigen testing and recall responses to tetanus toxoid, were recorded in our patients. Further studies are necessary to clarify the correlation between the expression of VEGF and immunity in patients with gastric carcinoma.

Antiangiogenic agents have received attention in clinical oncology as potential therapeutic agents (O'Reilly et al, 1994). TNP-470 is a synthetic analogue of fumagillin with strong anti-angiogenic activity; it inhibits tumour growth and metastasis in experimental models (Yamaoka et al, 1993; Yanase et al, 1993). A therapeutic blockade of the action of VEGF might improve prospects for immunotherapy, and it might also inhibit neovascularization of tumours.

In conclusion, expression of VEGF in gastric adenocarcinomas was associated with a decrease in the density of S-100 protein-positive DCs. Thus, VEGF might be associated with the progression of tumours and prognosis, not only via angiogenesis but also via avoidance of the induction of an immune response. Therapeutic blockade of the action of VEGF could conceivably provide a new treatment modality for patients with metastatic tumours.

### ACKNOWLEDGEMENT

This work was supported in part by grants from the Ministry of Education, Science, Sports and Culture of Japan and from the Uehara Memorial Foundation.

### REFERENCES

Ambe K, Mori K and Enjoji M (1989) S-100 protein-positive dendritic cells in colorectal adenocarcinomas: distribution and relation to the clinical prognosis. *Cancer* **63**: 496–503

Fontanini G, Vignati S, Boldrini L, Chiné S, Silvestri V, Lucchi M, Mussi A, Angeletti CA and Bevilacqua G (1997) Vascular endothelial growth factor is associated with neovascularization and influences progression of non-small cell lung carcinoma. *Clin Cancer Res* **3**: 861–865

Furukawa T, Watanabe S, Sato Y, Kodama T, Nakajima T and Shimosato Y (1984) Heterogeneity of histiocytes in primary lung cancer stained with anti-S100 protein, lysozyme and OKT 6 antibodies. *Jpn J Clin Oncol* **14**: 647–658

Gabrilovich DI, Chen HL, Girgis KR, Cunningham HT, Meny GM, Nadaf S, Kavanaugh D and Carbone DP (1996a) Production of vascular endothelial growth factor by human tumors inhibits the functional maturation of dendritic cells. *Nature Med* **2**: 1096–1103

Gabrilovich DJ, Nadaf S, Corak J, Berzofsky JA and Carbone DP (1996b) Dendritic cells in anti-tumor immune responses. II. Dendritic cells grown from bone marrow precursors, but not mature DC from tumor-bearing mice are effective antigen carriers in the therapy of established tumors. *Cell Immunol* **170**: 111–120

Inoue K, Ozeki Y, Suganuma T, Sugiura Y and Tanaka S (1997) Vascular endothelial growth factor expression in primary esophageal squamous cell carcinoma. *Cancer* **79**: 206–213

Japanese Research Society for Gastric Cancer (1995) *Japanese Classification of Gastric Carcinoma*. Kanehara, Tokyo

Kakeji Y, Maehara Y, Korenaga D, Tsujitani S, Haraguchi M, Watanabe A, Orita H and Sugimachi K (1993) Prognostic significance of tumor–host interaction in clinical gastric cancer: relationship between DNA ploidy and dendritic cell infiltration. *J Surg Oncol* **52**: 207–212

Katoh O, Tauchi H, Kawaishi K, Kimura A and Satow Y (1995) Expression of the vascular endothelial growth factor (VEGF) receptor gene, KDR, in hematopoietic cells and inhibitory effect of VEGF on apoptotic cell death caused by ionizing radiation. *Cancer Res* **55**: 5687–5692

Kekow J and Wiedemann GJ (1995) Transforming growth factor β: a cytokine with multiple actions in oncology and potential clinical applications. *Int J Oncol* **7**: 177–182

Maeda K, Chung YS, Ogawa Y, Takatsuka S, Kang SM, Ogawa M, Sawada T and Sowa M (1996) Prognostic value of vascular endothelial growth factor expression in gastric carcinoma. *Cancer* **77**: 858–863

Maehara Y, Tomisaki S, Oda S, Kakeji Y, Tsujitani S, Ichiyoshi Y, Akazawa K and Sugimachi K (1997) Lymph node metastasis and relation to tumour growth potential and local immune response in advanced gastric cancer. *Int J Cancer* **74**: 224–228

Matsuda H, Mori M, Tsujitani S, Ohno S, Kuwano H and Sugimachi K (1990) Immunohistochemical evaluation of squamous cell carcinoma antigen and S-100 protein-positive cells in human malignant esophageal tissue. *Cancer* **65**: 2261–2265

Miyake M, Taki T, Hitomi S and Hakomori S (1992) Correlation of expression of H/Le(y)/Le(b) antigens with survival in patients with carcinoma of the lung. *N Eng J Med* **327**: 14–18

Nakano T, Oka K, Arai T, Morita S and Tsunemoto H (1989) Prognostic significance of Langerhans' cell infiltration in radiation therapy for squamous cell carcinoma of the uterine cervix. *Arch Pathol Lab Med* **113**: 507–511

Nomori H, Watanabe S, Kaneya T, Nakajima T, Shimosato Y and Kameya T (1986) Histiocytes in nasopharyngeal carcinoma in relation to prognosis. *Cancer* **57**: 100–105

O'Reilly MS, Holmgren L, Shing Y, Chen C, Rosenthal RA, Mosesm M, Lane WS, Cao Y, Sage EH and Folkman J (1994) Angiostain: a novel angiogenesis inhibitor that mediates the suppression of metastases by a Lewis lung carcinoma. *Cell* **79**: 315–328

Toi M, Hoshina S, Takayanagi T and Tominaga T (1994) Association of vascular endothelial growth factor expression with tumor angiogenesis and with early response in primary breast cancer. *Jpn J Cancer Res* **85**: 1045–1049

Tsujitani S, Kakeji Y, Watanabe A, Kohnoe S, Maehara Y and Sugimachi K (1990) Infiltration of dendritic cells in relation to tumor invasion and lymph node metastasis in human gastric cancer. *Cancer* **66**: 2012–2016

Tsujitani S, Furukawa T, Tamada R, Okamura T, Yasumoto K and Sugimachi K (1987) Langerhans' cells and prognosis in patients with gastric carcinoma. *Cancer* **59**: 501–505

Yamaoka M, Yamamoto T, Masaki T, Ikeyama S, Sudo K and Fujita T (1993) Inhibition of tumor growth and metastasis of rodent tumors by the angiogenesis inhibitor O-(choroacetyl-carbamoyl) fumagillol (TNP-470; AGM-1470). *Cancer Res* **53**: 4262–4267

Yanase T, Tamura M, Fujita K, Kodama S and Tanaka K (1993) Inhibitory effect of angiogenesis inhibitor TNP-470 on tumor growth and metastasis of human cell lines in vitro and in vivo. *Cancer Res* **53**: 2566–2570

© Cancer Research Campaign 1998